# Screwing the helical chirality through terminal *peri*-functionalization

**DOI:** 10.3762/bjoc.22.14

**Published:** 2026-01-28

**Authors:** Devesh Chandra, Sachin, Upendra Sharma

**Affiliations:** 1 Chemical Technology Division, CSIR-IHBT, Palampur, HP 176 061, India; 2 Academy of Scientific and Innovative Research (AcSIR), Ghaziabad-201002, India

**Keywords:** catalysis, C–H functionalization, helical chirality

## Abstract

Helical chirality is an important, but underrated form of chirality than other types of chirality. Molecules with helical chirality impart a crucial role in several biological phenomena as well as in modern materials applications. Classically, the generation of chiral helicity in organic molecules relies on a ring extension by means of cycloaddition and related reactions. Recently, the *peri*-functionalization approach paved a new pathway for the generation of chiral helical molecules. In this article, we highlight the key advancements in these parallel approaches for the generation of helical chiral architectures.

## Introduction

Nature utilizes helical chirality for the inception of life. The double-helical structure of DNA and the helical conformations of proteins are key examples of the helical chirality. Inspired by these natural systems, chemists have developed methods to construct and control helicity in synthetic systems. These methods aim to achieve precise enantioselectivity, enabling the synthesis of helical molecules with specific handedness. In modern day science, helical chirality is gaining more and more importance in various fields such as drug design, optoelectronics, spintronics, diagnosis tool, biosensors and other forms of the materials sciences continuing its relevance to cutting edge research [1–2]. However, precise control and manipulation of helical chirality in synthetic systems remain technically challenging. This complexity may have led to underestimation of its potential, as research frequently focused toward other types of chirality, like point chirality [3], axial chirality [4], and planar chirality [5–7], often overshadowing the potential of helical chirality. Point chirality has major applications in medicinal chemistry and several drug molecules contain point chirality centers in their core structure [8]. Point chirality is the simplest form of chirality, and it is relatively easy to control and characterize during the synthesis of organic molecules. Due to these facts point chirality holds a central position among the different types of chirality. Axial chirality is another important type of chirality, where the chirality is controlled by restricted rotation among an axis. These types of molecules play an important role as chiral ligands in organic synthesis, i.e., BINOL, BINAP, etc. [9–11]. Another form of chirality, where chirality depends on a constrained substitution pattern along the plane of symmetry, is known as planar chirality, as seen in metallocene and cyclophanes. Molecules containing planar chirality have greater relevance in materials science and stereoselective transformations [12–13].

The fourth branch of the chiral tree is helical chirality. A helical system can undergo molecular to macroscopic systems due to its three-dimensional nature. Thus, helical systems, require more precise control of chirality compared to other forms of chirality. Helical chiral molecules have recently been found to display functional advantages, i.e., chirality-induced spin selectivity. These functionalities have great potential to transform in key areas such as drug discovery and modern diagnostic tools, as well as in distinct emerging fields like spintronics and optoelectronics [14].

Helicenes have found applications in chiral optoelectronics, i.e., as circularly polarized organic light emitting diodes (OLEDs), circularly polarized luminescence (CPL), and photonic devices, etc., due to their strong circular dichroism and circularly polarized luminescence [15–16]. Their well-defined three-dimensional chiral environment also makes helicenes useful in asymmetric synthesis, where they serve as chiral ligands and organocatalysts, delivering high enantioselectivities [17–18]. Their tunable HOMO–LUMO gaps and controlled π–π interactions, is key to the use of helicenes in molecular electronics and organic semiconductors as well. Helicenes function as active components in organic field effect transistors (OFETs), molecular wires, and chiral conductive materials [19–21]. Furthermore, helicenes are widely explored in chiroptical sensing, molecular recognition, and supramolecular self-assembly, enabling the construction of next generation responsive materials [22–23]. A very recent study also highlighted their potential application in biological interfaces [1], thus highlighting the versatility of helicenes in medicine along with next-generation materials and catalytic systems.

Due to the above-mentioned applications of chiral helicenes various synthetic approaches have been developed for the synthesis of chiral helical molecules and the most prevalent approaches include cycloaddition reactions [24], ring extensions [25], and related approaches [26–27]. However, some parallel and more sustainable approaches have emerged in recent years and C–H functionalizations are one of these approaches.

In the following section, we discuss the parallel approaches, i.e*., peri*-C–H functionalization, over the prevalent approach for the generation of helical chirality, which generally considers π extension. The *peri*-C–H functionalization approach provides direct access to a functional group installed in the terminal core of helicened with high level of stereocontrol, rather than merely increasing the number of fused rings in a helical system. The direct introduction of suitable functional groups at strategic positions can expand the structure diversity and enhance the utility of the parent helicene in diverse areas, thereby offering a more versatile alternative to classical helicene extension strategies.

## Discussion

**Figure 1:**
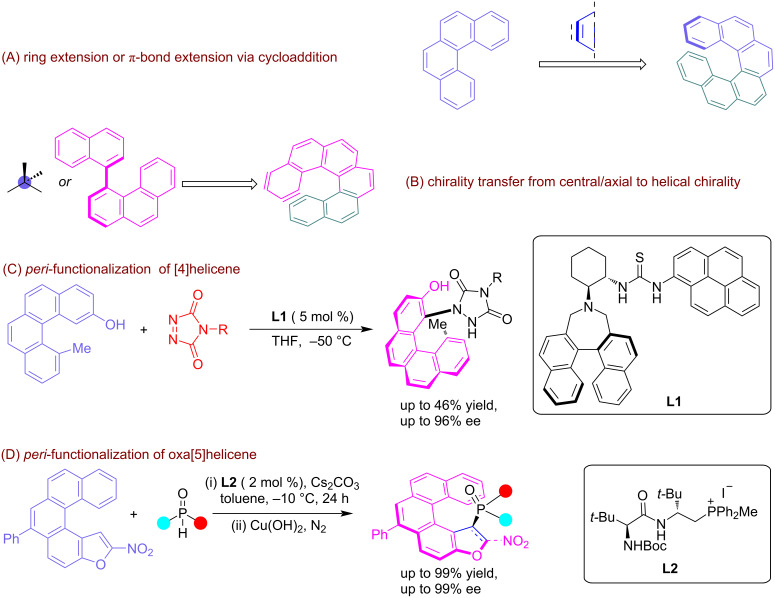
Representation of the general practices for the induction of helical chirality in organic scaffolds. A) Prelevant approach for the induction of helical chirality which majorly relies on the π extension of organic scaffolds via annulation/cycloaddition and B) central/axial to helical chirality conversion. C) Induction of helical chirality in [4]helicene via terminal *peri*-functionalization. D) Induction of helical chirality in [5]helicene via terminal *peri*-functionalization where a bulkier handle provides the required enantiomerization barrier to screw helicity.

Only a handful attempts have been made for the asymmetric synthesis of helical molecules using π conjugation extension [28–30]. This prevalent method for the generation of helical chirality primarily involves cycloaddition and central/axial to helical chirality transfer reactions [31] (Figure 1A and B). In addition to this, a parallel strategy also offers a promising approach to achieving configurationally stable helicenes via terminal *peri*-functionalization, where a bulkier handle provides the required enantiomerization barrier to screw helical chirality (Figure 1C and D).

**Scheme 1:**
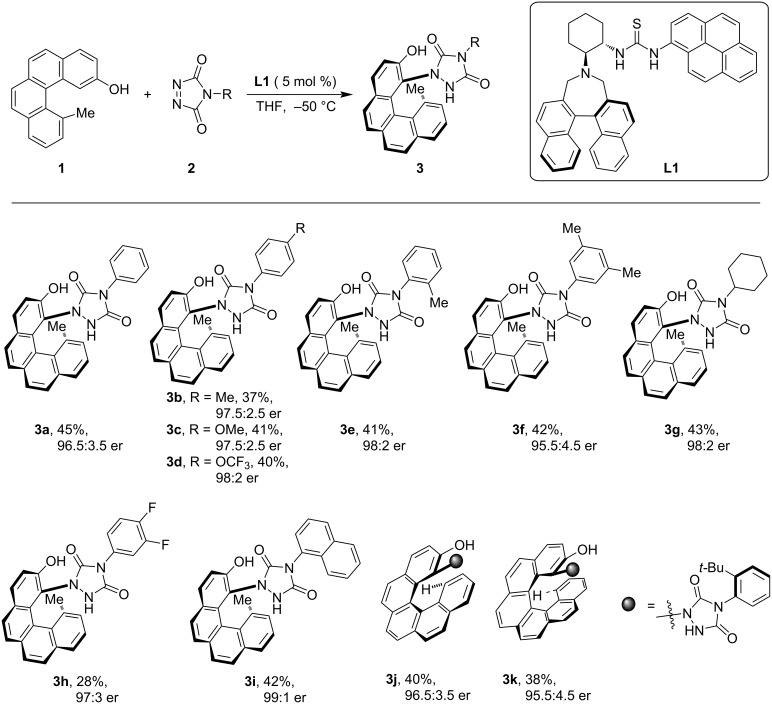
Asymmetric synthesis of carbohelicenes via *peri*-C–H functionalization.

A straight forward preparation of various carbohelicenes via an organocatalytic enantioselective hydroamination reaction of polyaromatic phenols with diazodicarboxamide derivatives through a catalytic kinetic resolution (KR) process was developed by Liu and co-workers [32]. The authors screened a range of organocatalysts, i.e., Takemoto’s thiourea catalyst, (*S*,*S*)-1,2-cyclohexanediamine-derived thioureas, cinchona alkaloid-derived thiourea, and squaramides to achieve the desired outcome of the reaction. After rigorous testing, (*S*,*S*)-1,2-cyclohexanediamine-derived catalyst **L1** was found to be the most effective organocatalyst to induce both the helical chirality and remote axial chirality during the functionalization of the [4]- and [5]helicenes **3a**–**k** with good yield and excellent enantioselectivity. Notably, [6]helicene **3k** was also readily produced, via kinetic resolution (Scheme 1).

**Figure 2:**
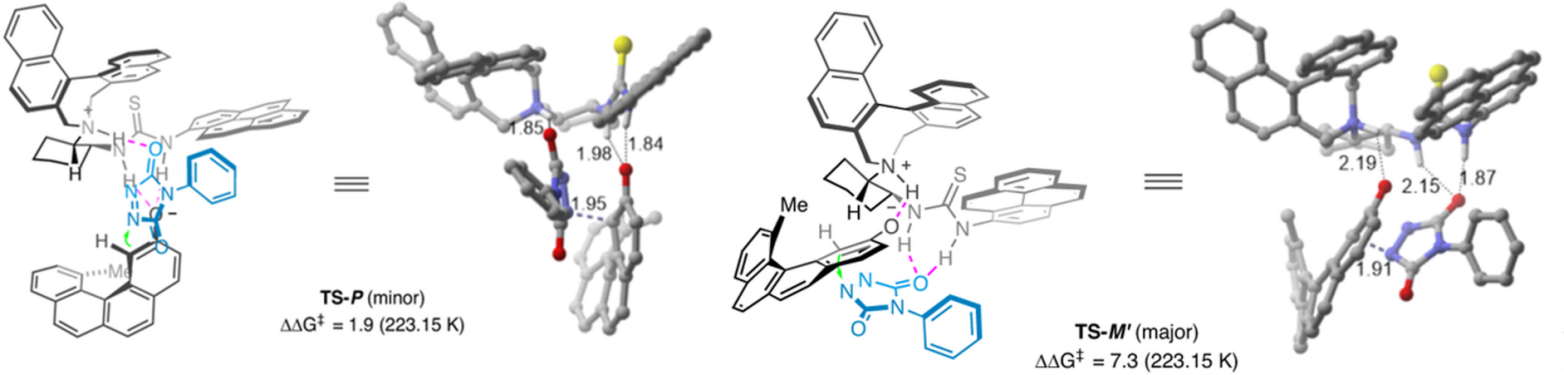
Stereomodel for *peri*-terminal functionalization of carbohelicene. (Figure 2 was reproduced from [32] (© 2024 X. Liu et al., published by Springer Nature, distributed under the terms of the Creative Commons Attribution 4.0 International License, https://creativecommons.org/licenses/by/4.0).

Computational studies suggested that hydrogen bonding and π interactions between the reactants and catalyst **L1** controlled the stereochemical output of the products **3**. The catalyst proximate both reactants to each other via hydrogen-bonding interaction, locking the relative orientation of the substrates producing the chiral [4]carbohelicene via *peri*-terminal functionalization (Figure 2).

**Scheme 2:**
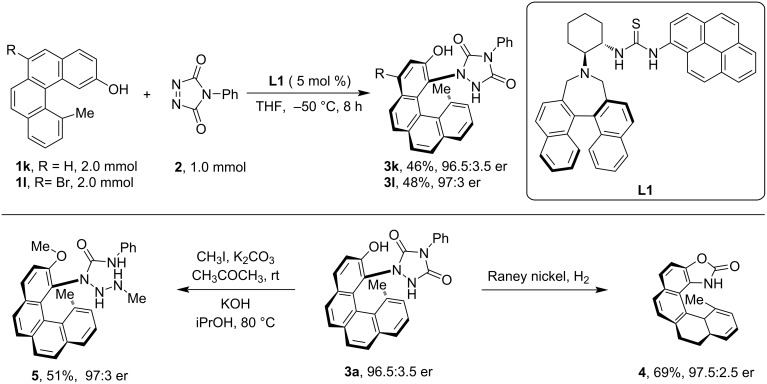
Scale-up synthesis and post-synthetic application of chiral helical product **3**.

To examine the utility of the developed protocol, the reaction was scaled up to a 2 mmol scale and the product was further transformed into representative derivatives. Notably, the reaction performed well in the scale-up demonstration and the post-synthetic transformations proceeded smoothly without hampering the enantiomeric excess (Scheme 2).

**Scheme 3:**
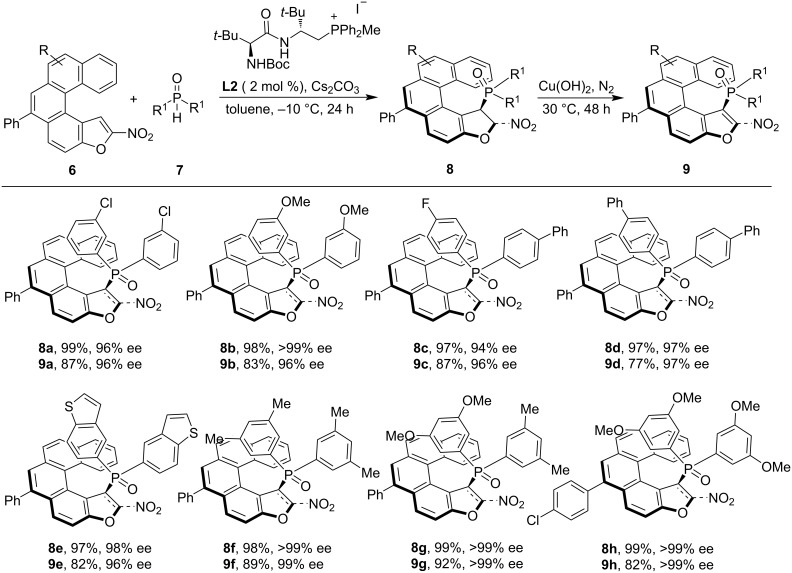
Asymmetric peri-C–H functionalization of nitro-substituted oxa[5]helicenes.

Another report applying *peri*-C–H functionalization for the synthesis of helical chiral entities was reported by Wang and co-workers. They reported an organocatalyzed asymmetric synthesis of phosphorus-containing chiral helicenes enabled by dynamic kinetic resolution using copper and peptide-mimetic phosphonium salts, i.e. amino acid-derived phosphonium iodide and bromide as catalysts [33]. A domino reaction was developed, beginning with a PPS **L2**-catalyzed Michael addition of phosphine oxides **7** to nitro-substituted oxa[5]helicenes **6**, followed by a copper-promoted aromatization. This sequence efficiently produced phosphorus-containing oxa[5]helicene derivatives **8** in high yields and with excellent enantioselectivities. The catalytic system operated under mild conditions within a practical timeframe and demonstrated broad substrate tolerance, providing a range of chiral helicenes. Notably, unsymmetrical phosphine oxides also underwent smooth Michael addition, with excellent enantiomeric excess and diastereomeric ratio (Scheme 3).

**Scheme 4:**
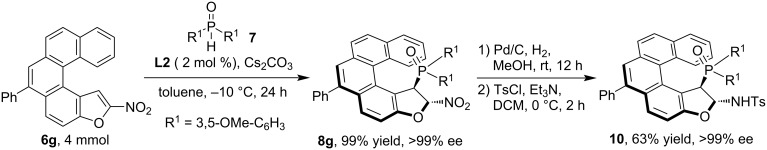
Scale-up synthesis and post-synthetic application of chiral helical product **8g**.

The developed protocol was also tested for up-scale synthesis and post-synthetic transformations. Notably, the reaction worked well at a 4.0 mmol scale providing product **8g** in quantitative yield and >99% ee. This product was further reduced to the corresponding amine followed by *N*-tosylation, providing product **10** in 63% yield without altering the enantiomeric excess (Scheme 4).

**Scheme 5:**
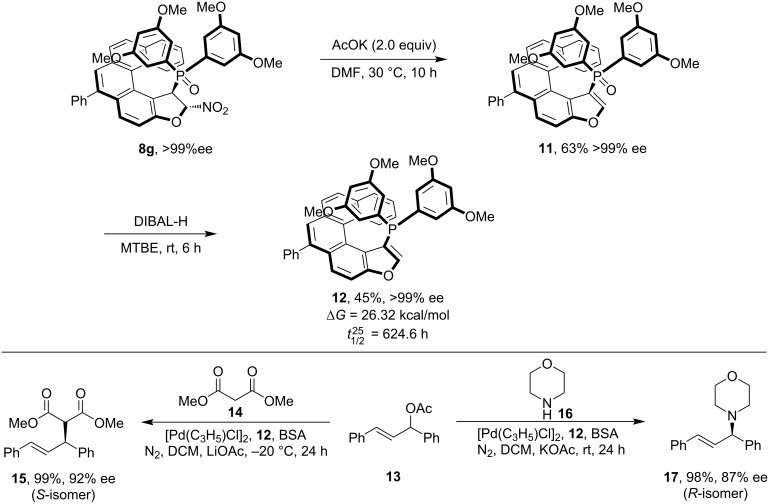
Post-synthetic transformation of product **8g** to chiral phosphine ligand **12** and its application in Pd-catalyzed reactions.

Additionally, product **8g** was converted into the chiral phosphine ligand **12** while fully preserving its helical chirality and successfully applied in palladium-catalyzed nucleophilic substitution reactions (Scheme 5).

**Figure 3:**
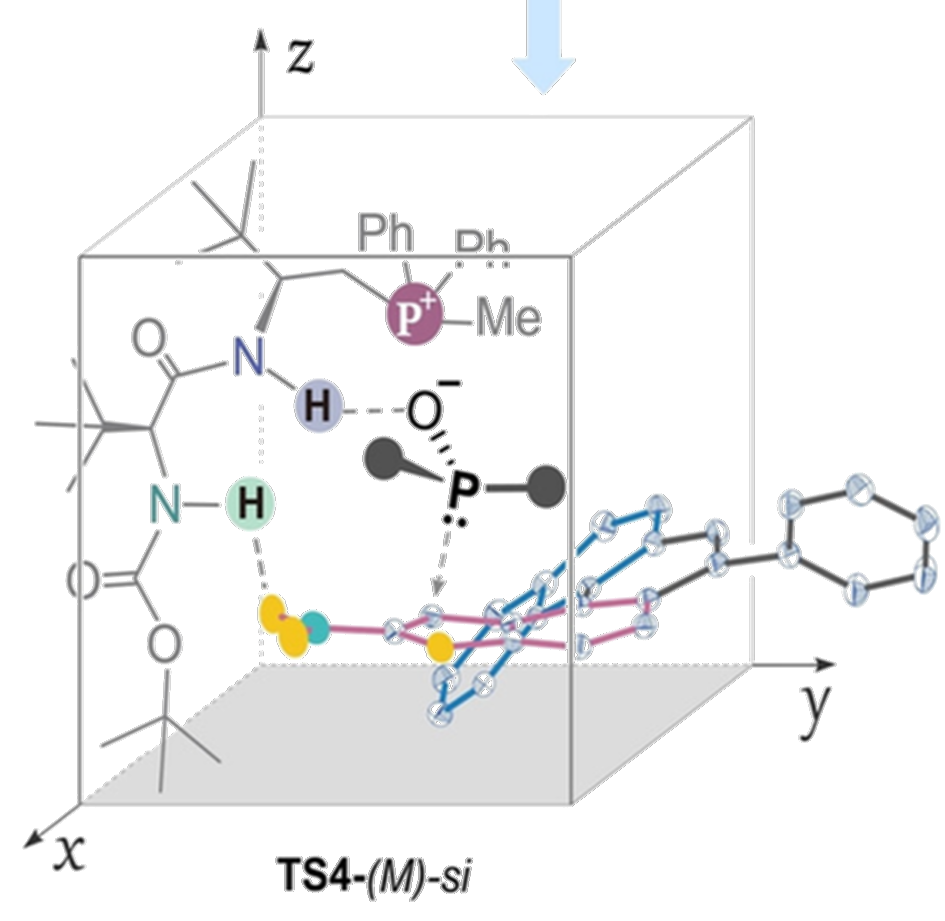
Stereomodel for the asymmetric *peri*-C–H functionalization of oxa[5]helicene. (Figure 3 was reproduced from [33], J.-H. Wu et al., *Angew. Chem., Int. Ed.*, with permission from John Wiley and Sons. Copyright © 2023 Wiley-VCH GmbH. This content is not subject to CC BY 4.0)

A stereochemical model was proposed for the phospha-Michael addition, indicating that enantioselectivity was primarily influenced by steric effects arising from hydrogen bonding and ion pairing between the peptide mimetic phosphonium salt (PPS)-activated phosphine oxide and the nitro-functionalized oxa[5]helicene. This ion-pairing interaction was also studied using NMR titration and Job’s plot analysis. The transition state depicted in Figure 3 was found to be most favorable providing the product with *M*-configuration.

Looking ahead, these strategies may also be applicable to other polyaromatic frameworks beyond specific helicenes for the construction of helically chiral structures.

## Outlook

Despite its potential, this strategy has faced significant challenges in the context of catalytic enantioselective synthesis of functionalized helicenes and their heteroanalogues. These challenges include: (1) the limited availability of suitable substrates, (2) substantial steric hindrance from the terminal ring, making it difficult to introduce substituents at the required positions onto helical precursors, and (3) the complex nature of controlling helical chirality, a nanoscale phenomenon that requires the design of advanced catalysts to achieve high enantioselectivity. Although the parallel approach is still in its infancy, it holds immense promise. With the continued and widespread efforts of synthetic chemists, we anticipate that the existing challenges will be surmounted, paving the way for the development of innovative approaches and new materials. Such advancements are expected to unlock the full potential of chiral helicenes.

## Data Availability

Data sharing is not applicable as no new data was generated or analyzed in this study.
